# The Adsorption of H_2_ and C_2_H_2_ on Ge-Doped and Cr-Doped Graphene Structures: A DFT Study

**DOI:** 10.3390/nano11010231

**Published:** 2021-01-16

**Authors:** Yiming Liao, Ruochen Peng, Shudi Peng, Wen Zeng, Qu Zhou

**Affiliations:** 1 College of Engineering and Technology, Southwest University, Chongqing 400715, China; ymliao614@163.com (Y.L.); ruochenpeng@163.com (R.P.); 2 Chongqing Electric Power Research Institute, State Grid Chongqing Electric Power Company, Chongqing 401123, China; pengshudi@163.com; 3 College of Materials Science and Engineering, Chongqing University, Chongqing 400044, China

**Keywords:** Ge doping, Cr doping, graphene, oil dissolved gases, DFT calculations

## Abstract

In order to find an excellent sensing material for dissolved gases in transformer oil, the adsorption structures of intrinsic graphene (IG), Ge-doped graphene (GeG), and Cr-doped graphene (CrG) to H_2_ and C_2_H_2_ gas molecules were built. It was found that the doping site right above C atom (T) was the most stable structure by studying three potential doping positions of the Ge and Cr atom on the graphene surface. Then, the structural parameters, density of states, and difference state density of these adsorption systems were calculated and analyzed based on the density functional calculations. The results show that the adsorption properties of GeG and CrG systems for H_2_ and C_2_H_2_ are obviously better than the IG system. Furthermore, by comparing the two doping systems, CrG system exhibits more outstanding adsorption performances to H_2_ and C_2_H_2_, especially for C_2_H_2_ gas. Finally, the highest adsorption energy (−1.436 eV) and the shortest adsorption distance (1.981 Å) indicate that Cr-doped graphene is promising in the field of C_2_H_2_ gas-sensing detection.

## 1. Introduction

As one of the main pillars of the current economy, electric energy is gradually accelerating the pace of its intelligent construction, and the scale is also expanding. The oil-immersed transformer, as the key hub of a power system, undertakes the task of power transmission and transformation of the whole power grid, and its operation condition will directly affect the safety of the power network and users [1,2,3,4]. However, insulation faults like partial discharge and partial overheating inevitably exist during oil-immersed transformer long running process [5,6,7,8]. These internal faults can cause the generation of various fault characteristic gases, such as H_2_ [9], CH_4_ [10], C_2_H_2_ [11], etc. Therefore, detecting typical dissolved gases have great importance to guarantee the normal operation of a power transformer [12,13,14].

With the discovery of two-dimensional materials, graphene material has captured widespread attention for its excellent performances and considerable applications [15,16,17]. Nevertheless, there is the weak adsorption capacity of the intrinsic graphene to gas molecules, which limits its application in gas-sensing respects [18,19,20,21]. To address this issue, researchers have built new graphene substrates via doping different atoms, such as Pt [22], In [23], Co [24], etc., in which metal atoms with a highly active doped-on graphene surface greatly improves its adsorption properties [25,26]. For instance, Shukri et al. researched the interaction mechanism of NO and CO on Pd-decorated graphene via first-principles study [27]. Zheng et al. analyzed the CO_2_ adsorption behavior on doped (B, P, N, Al) graphene by density functional theory (DFT) calculations and indicated that metal doping can be an ideal method to enhance the performances of a graphene-based gas sensor [28]. Gao et al. discussed the structural parameters of an SO_2_ molecule on intrinsic graphene and transition metal (Ni, Pd) -doped graphene, suggesting that the latter exhibits stronger adsorption [29]. Hence, metal-doped graphene might be a potential sensing material for detecting oil-dissolved gases.

Chromium (Cr) has received considerable investigation with its high electrical conductivity and excellent ductility as a transition metal (TM) atom [30]. Germanium (Ge) has some interesting electrochemical properties due to its position between metallic and nonmetallic elements [31]. Until now, the two kinds of metal atoms have been designed in different materials by investigators through various methods to achieve excellent properties in diverse fields [32,33,34]. For instance, Zhou et al. exhibited that Cr-decorated graphene could enhance gas adsorption of a formaldehyde (H_2_CO) molecule in the aspect of atmospheric environment [35]. Wang et al. found that Cr doping CoFe-layered double hydroxides might improve adsorption property to water and urea molecule in an electro catalytic field [36]. Gecim et al. compared the gas-sensitive properties of methanol on Ge-doped and Ga-doped graphene and signified the more excellent adsorption performance of Ge-doped graphene in terms of methanol gas sensing [37]. However, few pieces of literature have been studied using intrinsic graphene (IG), Ge-doped graphene (GeG), and Cr-doped graphene (CrG) as gas-sensing materials for detecting gases dissolved in transformer oil. Therefore, in this study, the adsorption properties of Ge-doped and Cr-doped graphene are investigated to H_2_ and C_2_H_2_ molecules based on density functional theory (DFT). To analyze and compare the sensing mechanism and adsorption capacity of three kinds of graphene models, their geometric structures, adsorption distances, bond lengths, adsorption energies ($E_{ads}$), charge transfers ($Q_{t}$), density of states (DOS), and atomic orbitals were calculated. The consequences of research provide theoretical guidance for detection of H_2_ and C_2_H_2_ gas molecules.

## 2. Computation Methods

Density functional calculations between gas molecules and three graphene-based materials were carried out in Dmol3 module of Materials Studio (MS) [38,39,40]. To guarantee the reliability of calculation, the corresponding issues of exchange energy were solved by generalized gradient approximation (GGA) with the functional Perdew–Burke–Ernzerhof (PBE) [41]. In this paper, the further calculation was implemented with the double numerical plus polarization (DNP) to obtain more accurate results [42]. The periodic box conditions (PBC) were employed to describe the 2D structure on the metal-doped graphene surface, and the graphene-based lattice parameters were a = 9.84 Å, b = 9.84 Å, c = 15 Å. The DFT semi-core pseudopotential (DSPP) and the iterative subspace direct inversion (DIIS) were respectively utilized to deal with core electrons and accelerate the convergence of Self-Consistent Field (SCF) charge density [43]. Simultaneously, given the important effect of weak van der Waals interaction in gas molecules, the Grimme (DFT-D2) algorithm was used to correct the dispersion energy [44]. The values of 1 × 10^−5^ Ha, 2 × 10^−3^ Ha/Å, and 5 × 10^−3^ Ha were set for energy convergence accuracy, maximum displacement, and stress, respectively. The convergence accuracy of the Self-Consistent Field (SCF) and the k-points of the Brillouin zone were set to 1 × 10^−6^ Ha and 6 × 6 × 1. The setting of density of state (DOS) in Dmol3 calculation properties was selected to qualitatively analyze the electronic structure of the material by outputting the charts of the total density of state (TDOS) and partial density of state (PDOS). All of the above calculations were considered Spin polarization effect [45].

The adsorption energy ($E_{ads}$) of gas (H_2_ and C_2_H_2_) on three kinds of graphene-based material is defined in Equation (1).

(1) $$E_{ads}=E_{gas-substrate}-E_{substrate}-E_{gas}$$

In which, $E_{gas-substrate}$ presents the total energies of gas adsorbed graphene system. $E_{substrate}$ and $E_{gas}$ are the energies of the graphene-based system before gas adsorption and a single gas molecule, respectively [46]. The negative value of $E_{ads}$ shows the spontaneity of reaction and the exothermic process of gas adsorption [47]. Moreover, the change transfer $Q_{t}$ between the graphene system and gas molecules is defined in Equation (2)

(2) $$Q_{t}=Q_{a}-Q_{b\;}$$

where $Q_{a}$ and $Q_{b}$ were the number of charges after adsorption carried by the gas and the net carried charge of isolated gas molecule, respectively. The positive value of $Q_{t}$ represents the charge transfer from gas molecules to the graphene-based system [48].

## 3. Results and Discussions

### 3.1. Structures of H_2_ and C_2_H_2_ Molecules and IG

**Figure 1:**
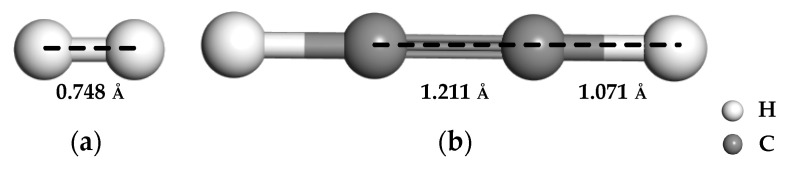
The structures of H_2_ (**a**) and C_2_H_2_ (**b**).

**Figure 2:**
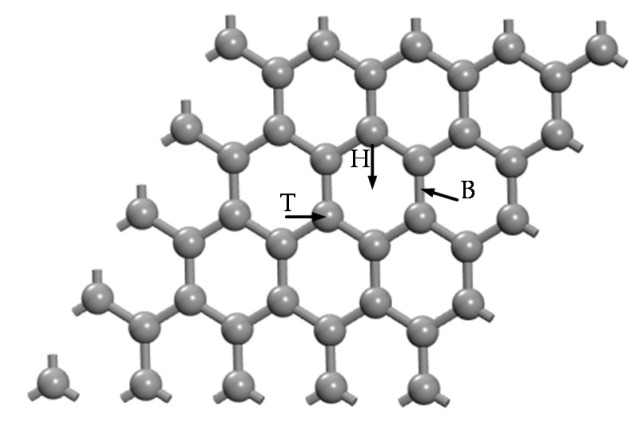
Three potential doping sites of graphene.

The models of H_2_ and C_2_H_2_ gas molecules were firstly established, and their optimized structures are presented in Figure 1. The bond length of carbon-carbon triple and carbon-hydrogen is 1.211 Å and 1.071 Å in C_2_H_2_ molecule, respectively. The IG was established with a 4 × 4 × 1 (32 atoms) supercell with the vacuum slab of 20 Å to avoid layer interaction and the slab position of 10 Å to build a two-dimensional graphene monolayer. The models of GeG or CrG were constructed by different sites doping with a Ge or Cr atom. To obtain the steadiest doped structure, the three potential sites of metal-doped graphene structure are discussed, including the doping site right above C atom (T), the hollow site in the positive center of graphene lattice (H), and the bridge site at midpoint the two C atoms (B), which are shown in Figure 2. The binding energy ($E_{b}$) formula of metal atoms doped on intrinsic graphene is defined as follows: Equation (3) is suitable for calculating the substitution doping of C atoms on the intrinsic graphene [49], and Equation (4) is used to calculate the surface doping of intrinsic graphene [50]. In which, $E_{metal}$, $E_{C}$, $E_{\mathrm{graphene}}$ and $E_{metal-\mathrm{graphene}}$ are the total energies of the single metal atom, the replaced C atom, the intrinsic graphene, and graphene system after doping metal atom, respectively. Remarkably, the binding energy is only studied from the perspective of the electronic component and the above formulas are applicable to the doping of Ge and Cr metal atoms on the surface of graphene.

(3) $$E_{b}=E_{metal-\mathrm{graphene}}-E_{metal}-E_{\mathrm{graphene}}+E_{C}$$

(4) $$E_{b}=E_{metal-\mathrm{graphene}}-E_{metal}-E_{\mathrm{graphene}}$$

### 3.2. Ge and Cr Doping on Graphene

**Figure 3:**
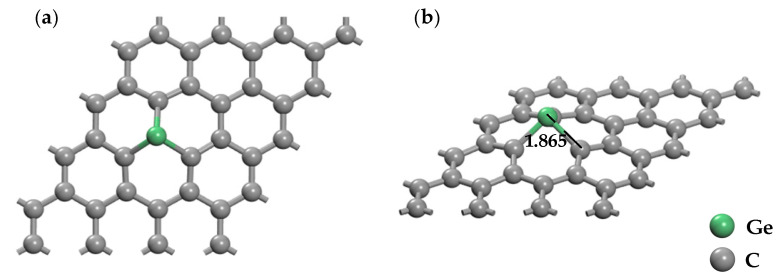
The optimized structure of Ge-doped graphene (GeG) at T doping site (**a**) top view (**b**) side view.

By comparing the detailed calculated parameters information in Table 1, the most stable structure for Ge-doped graphene is the T site model as shown in Figure 3. After geometry optimization, the Mulliken analysis shows that there is an obvious charge transfer (−0.525 e) in G_T_ doping structure, while the charge transfers in G_H_ and G_B_ doping structures are 0.135 e and 0.032 e, respectively. The C atom of graphene and the Ge atom form the C-Ge bond by the shortest length of 1.865 Å in the G_T_ doping structure, which is much shorter than that in G_H_ (2.119 Å) and G_B_ (2.279 Å) doping structures. Remarkably, the larger bind energy of 4.725 eV is consumable to form new Ge-C bonds, because the substitution doping of the T site destroys the internal structure of graphene.

**Figure 4:**
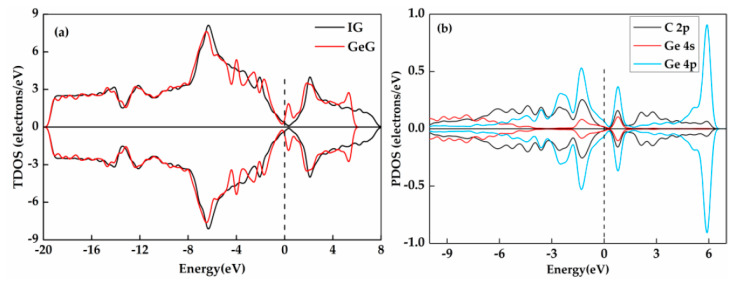
The total density of state (TDOS) (**a**) and partial density of state (PDOS) (**b**) of GeG system at T doping site.

The total density of state (TDOS) and partial density of state (PDOS) of GeG in G_T_ doping structure are shown in Figure 4. It can be found that the TDOS of the graphene after doping has a distinct rise amplitude near the Fermi level and the new peaks appear near −1 eV. The PDOS (Figure 4b) of each atomic orbital is investigated to further discuss the interaction between graphene and doped-Ge atom. According to the PDOS diagram, the variation of TDOS around the Fermi level is mostly caused by hybridization of C-2p and Ge-4p orbitals. Furthermore, the overlap of C-2p orbital and Ge-4p orbital is evident at −1 eV, manifesting that the doping of Ge atom may be one of the reasons for the appearance of the new peaks of TDOS here. The high peak of Ge-4p orbital around 6 eV indicates the rise of TDOS curve here is mainly contributed by it. Therefore, the Ge atom and graphene have a stable adsorption structure by doped T site.

**Figure 5:**
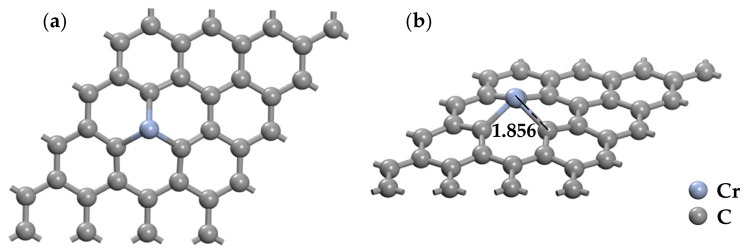
The optimized structure of Cr-doped graphene (CrG) at T doping site (**a**) top view (**b**) side view.

As shown in Table 2, the parameters of different doping structures are different when a Cr atom approaches the surface of graphene with different sites. The Mulliken charge analyzes electron transfer between Cr and graphene in the three potential doping sites. The doping structures of G_H_ and G_B_ show that the *Q_t_* is 0.211 e and 0.236 e, while the charge transfer value in the G_T_ doping structure is −0.252 e, which is higher than that of other structures. Moreover, the Cr-C bond (1.856 Å) of the G_T_ doping structure is shortest compared with that of G_H_ (2.031 Å) and G_B_ (1.386 Å), but the consumable bind energy (4.732 eV) of the G_T_ site is larger to form the new Cr-C bonds. The most stable doping configurations obtained by comparing various geometric parameters are depicted in Figure 5.

**Figure 6:**
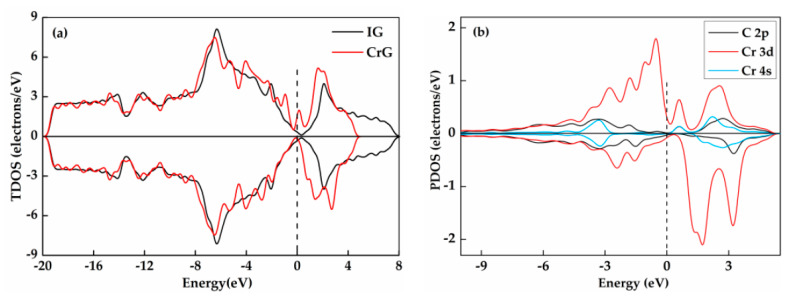
The TDOS (**a**) and PDOS (**b**) of CrG system at T doping site. The “+” for alpha-spin, “−” for beta-spin in the TDOS plot.

Spin-polarized total DOS for IG and CrG in G_T_ doping structure is depicted in Figure 6. As shown in Figure 6a, the TDOS curve of the CrG system is closer to the Fermi level. In addition, the charge density increases significantly in this area. Compared with the TDOS of IG, the eigenstates of both spin-up and spin-down contributions of the TDOS exhibit obviously different near the Fermi level in CrG system, which indicates that the doping of Cr atom changes the non-magnetism of the system. Both alpha-orbital of Cr-3d and Cr-4s orbitals have valley near the Fermi level, while only the beta-orbitals of Cr-3d has peak here, as shown in Figure 6b. Additionally, these obvious asymmetries of spin-up and spin-down densities of states of Cr-3d and Cr-4s orbitals near the Fermi level can further prove that the doping of Cr leads to the change of non-magnetism of system. Moreover, the maximum value of the valence band and the conduction band is mainly affected by Cr-3d orbital. The larger overlap parts of the C-2p orbital, Cr-3d orbital, and Cr-4s orbital are observed around the 0 eV, signifying the close interaction between Cr atom and graphene due to the strong hybridization of these orbitals.

According to the above analysis results, the most stable structures of doped graphene-based materials are doped by T sites, which are C atoms of the graphene structure substituted with Ge or Cr atoms. The metals doping has a strong effect on the electronic structural performances of graphene. Then, the adsorption effects of the three materials of graphene-based to oil-dissolved gas molecules are investigated based on the stable doped structures, especially the adsorption effects of H_2_ and C_2_H_2_ molecules.

### 3.3. H_2_ Adsorbed on IG, GeG, and CrG Systems

**Figure 7:**
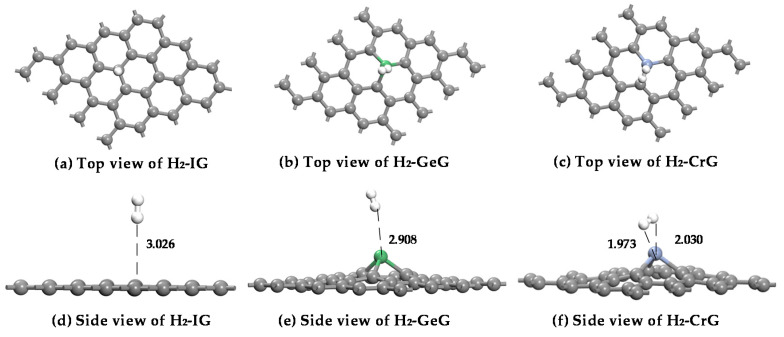
The optimal structures of H_2_ adsorbed on IG (**a**,**d**), GeG (**b**,**e**), and CrG (**c**,**f**) systems.

The adsorption models of H_2_ on intrinsic graphene, Ge-graphene, and Cr-graphene have been established, and the structures after optimization are exhibited in Figure 7**.** Comparing the three adsorption models, a H_2_ molecule tends to vertically close to the surface of IG and GeG systems, to form a stable adsorption structure, while H_2_ molecule adsorbs parallelly on Cr atom in CrG system. Table 3 displays that the adsorption energy of IG, GeG, and CrG systems is −0.153 eV, −0.117 eV, −0.390 eV. The preliminary results display that the adsorption energy of CrG is larger than that of IG and GeG systems, probably because of the doped Cr atom with stronger surface activity. Besides, the adsorption parameters of H_2_ molecule have small change in IG and GeG systems. Nevertheless, there are distinct variations of structural parameters in the CrG system, as the adsorption distance is obviously smaller between a H_2_ molecule and graphene-based material than that in IG (3.026 Å), and the H_2_-adsorbed Cr-graphene exits significant charge transfer (0.052 e). Therefore, these findings suggest that the CrG system is the steadiest adsorption structure to H_2_ molecule in metal-doped graphene.

**Figure 8:**
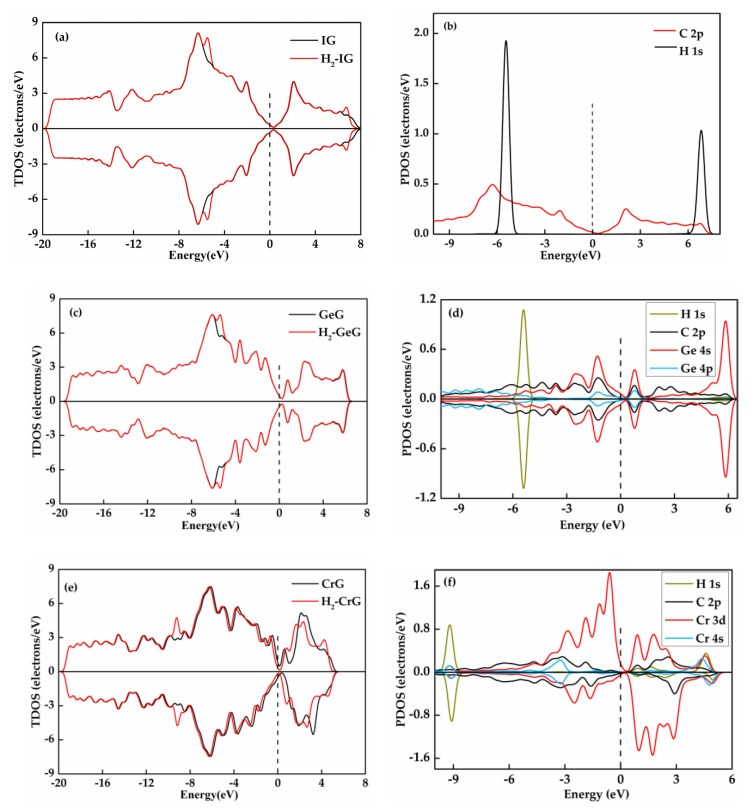
The TDOS and PDOS of H_2_ adsorbed on IG (**a**,**b**), GeG (**c**,**d**), and CrG (**e**,**f**).

To further investigate the adsorption behavior of H_2_ on IG, GeG, and CrG systems, the DOS diagrams of various systems are analyzed. According to the TDOS and PDOS of IG system displayed in Figure 8a,b, the H_2_ adsorption has a small influence on the DOS curve due to less hybridization between H-1s orbital and C-2p orbital. Figure 8c,d present the TDOS and PDOS of GeG system. It is found that only the peak near −5 eV changes obviously after H_2_ adsorption, while the other distribution of TDOS diagram is almost unchanged in GeG system, and the varied DOS mainly caused by the 1s orbital of H atoms. As for H_2_ adsorption, the total DOS of the adsorption system does not change distinctly near the Fermi level in the IG and GeG system. Thus, the introduction of H_2_ molecules has little effect on electron properties of intrinsic graphene and Ge-graphene. As the TDOS of CrG depicted in Figure 8e, the curve moves to left overall. Besides, the curve changes obviously around the Fermi level, resulting in the electron transferred easily from valence band to conduction band. Figure 8f shows the H-1s, Cr-4s, and Cr-3d orbitals have considerable overlaps around −9 eV, and H-1s orbital hybridizes slightly with Cr-4s orbital around the Fermi level, indicating that the occurrence of strong interaction between H_2_ molecule and Cr-graphene mainly contributes by the hybridization of H-1s and Cr-4s orbitals, while Cr-3d orbital may be main reason for the alpha-spin and beta-spin asymmetry of TDOS. In brief, CrG system shows a stronger adsorption to a H_2_ molecule than that of IG and GeG systems, because of the clearly varied DOS of CrG system during the adsorption process.

**Figure 9:**
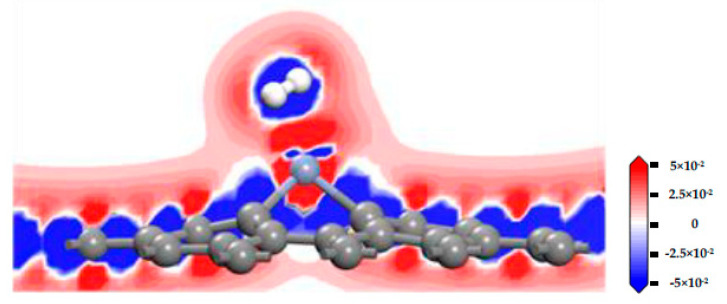
The charge density difference of CrG system.

Figure 9 displays the charge density difference of the CrG system, and the getting and losing of the electron can be seen in the red and blue areas, respectively. H_2_ molecule and Cr atom are surrounded by blue and red, respectively, which indicates that Cr atom obtains electrons from the H_2_ molecule. This phenomenon further confirms that charges transfer from H_2_ molecule to CrG, which is uniform with the results of the *Q_t_* (0.052 e). In addition, a certain amount of interaction exists because of the continuous electron area between H atom and Cr atom.

### 3.4. C_2_H_2_ Adsorbed on IG, GeG, and CrG Systems

**Figure 10:**
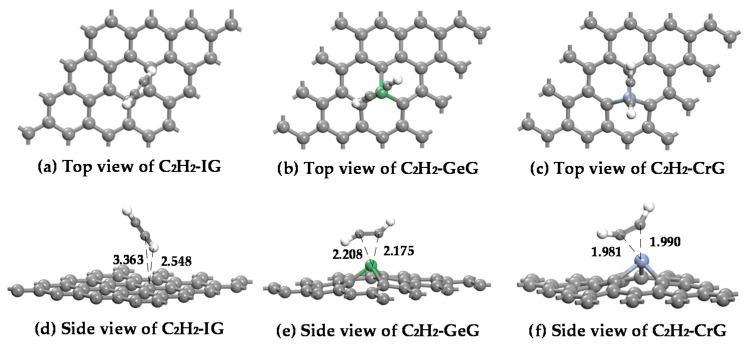
The optimized structures of C_2_H_2_ adsorbed on IG (**a**,**d**), GeG (**b**,**e**), and CrG (**c**,**f**) systems.

The adsorption structures of IG, GeG, and CrG systems to C_2_H_2_ in top and side views are shown in Figure 10, and Table 4 lists the corresponding adsorption parameters of IG, GeG, and CrG systems to C_2_H_2_.

For C_2_H_2_ adsorption on the IG system, the C_2_H_2_ molecule is far from the graphene surface because of the weak interaction. Table 4 shows that the *E_ads_* and *Q_t_* of the IG system are only −0.066 eV and −0.008 e, which are far smaller than those of GeG and CrG systems. For C_2_H_2_ adsorption on the GeG system, the C_2_H_2_ molecule is distinctly deformed after the captured C atom of C_2_H_2_ molecule by the Ge atom, while C_2_H_2_ is adsorbed on the top site of the C atom by an H atom in the IG system. It is notable that the C_2_H_2_ molecule still exhibits a planar configuration after capturing by graphene monolayer, indicating the nonactivation of C_2_H_2_ molecule during the adsorption. By comparing the adsorption parameters of GeG structures in Table 4, the distance between C atoms of C_2_H_2_ molecule and Ge-graphene is shorter, which indicates that the C_2_H_2_ molecule is more likely to be absorbed on Ge-graphene surface by C atom. For C_2_H_2_ adsorption on Cr-graphene, a C_2_H_2_ molecule adsorbs on Cr atom with C-Cr bond, and the bond angle of the C_2_H_2_ molecule changes significantly in the adsorption process. The C-H band was a little elongated from 1.071 Å to 1.089 Å, resulting from the impact of strong adsorption of Cr-C bonds. The adsorption energy of Cr-graphene reaches −1.436 eV, the charge transfer amounts 0.079 e, and the distance is 1.981 Å. Comparing to intrinsic graphene and Ge-graphene, Cr-graphene has the best adsorption energy, largest charge transfer, and shortest distance.

In conclusion, considering the energy and structures of IG, GeG, and CrG adsorption systems, the adsorption of C_2_H_2_ molecule on the intrinsic graphene and Ge-graphene is relatively weaker, while Cr-graphene exhibits strong adsorption capacity owing to the huge adsorption energy (−1.436 eV). Furthermore, CrG system should be the most stable structure due to the excellent adsorption properties.

**Figure 11:**
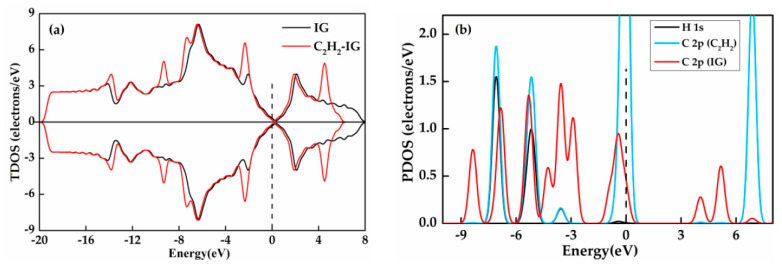

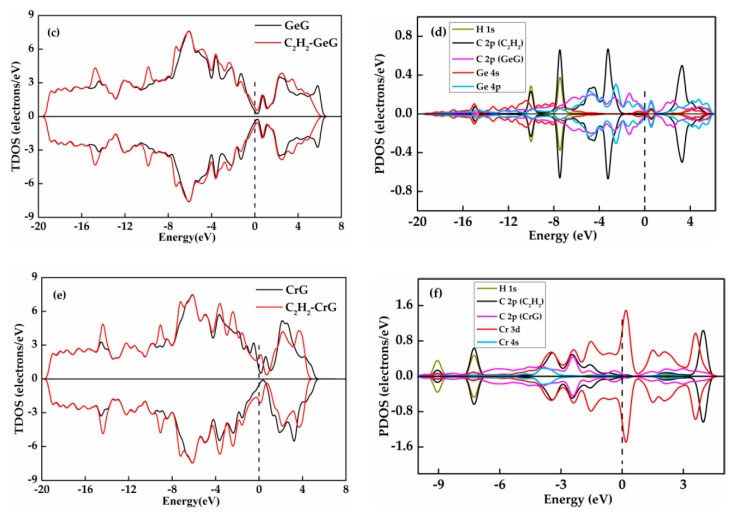
The TDOS and PDOS of C_2_H_2_ adsorbed on IG (**a**,**b**), GeG (**c**,**d**) and CrG (**e**,**f**).

The TDOS and PDOS distributions for C_2_H_2_ system are displayed in Figure 11. It can be found that the two TDOS (Figure 11a) curves are overlapped near the Fermi level and the PDOS (Figure 11b) diagram of H-1s and C-2p orbitals are hardly hybridized in this area, implying the weak interaction between the C_2_H_2_ molecule and intrinsic graphene and the few charge transfers of adsorption. For the GeG system depicted in Figure 11c, the overall curve of the DOS is clearly closer to Fermi level after C_2_H_2_ molecule adsorption, suggesting the notable change of charge density and the easy transfer of electron distribution from valence to conduction band. Figure 11d displays that the overlapping peaks of H-1s, C-2p, Ge-4s, and Ge-4p orbitals appear near the Fermi level, manifesting the strong hybridization of these orbitals. Besides, the high amplitudes of C-2p orbital of C_2_H_2_ near −7 eV, −3 eV, and 3 eV imply that the appearance of the new peak of TDOS at these place may be caused by C_2_H_2_ adsorption. Figure 11e shows that the TDOS of CrG system goes from asymmetric to symmetric in the spin up and spin down channels at some energies, indicating that the C_2_H_2_ adsorption turns magnetic Cr-graphene into a non-magnetic system. From Figure 11f, the 2p orbital of C atom, the 3d and 4s orbitals of Cr atom have a large range of strong overlap at −2 eV to −4 eV, and the PDOS diagram of H-1s, C-2p, Cr-3d, and Cr-4s orbitals basically overlap from −7 eV to −9 eV, signifying the occurrence of strong hybridization.

**Figure 12:**
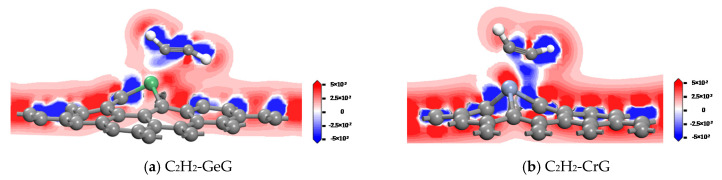
The charge density difference of the C_2_H_2_ molecule on GeG (**a**) and CrG (**b**) systems.

By analyzing the charge density difference as shown in Figure 12, the electron transfer mechanism of GeG and CrG system is researched. Figure 12a shows the obvious contact of the electron occurrence region between the C_2_H_2_ molecule and Ge atom, which infers a strong interaction between them because of a certain amount of transferred electron. A direct continuous electron region is found in Figure 12b, indicating that part of electron transfers between C and H atoms. In addition, the Cr atom is surrounded distinctly by red areas, while the C atom of C_2_H_2_ is surrounded by blue areas. This phenomenon suggests that a mass of electrons transfer from the C_2_H_2_ molecule to CrG system. The findings show that a strong chemical interaction appears between C_2_H_2_ and CrG, and results in the appearance of a new Cr-C bond. The above conclusions are uniform with the consequences of the Mulliken analysis.

## 4. Conclusions

In this paper, the adsorption abilities of IG, GeG, and CrG systems towards two oil-dissolved gases (H_2_ and C_2_H_2_) were investigated via a detailed DFT study. The most stable structure was explored by studying structural and electronic properties of various adsorption models. The adsorption energy, charge transfer, density of state, and other structural parameters have been employed to discuss the adsorption mechanism. It is found that both Ge doping and Cr doping by T site have most stable doping structure through researching three typical doping structures. By constructing and optimizing different adsorption models of gas molecules to IG, GeG, and CrG systems, H_2_ closes to Ge and Cr atom through H atom, but C atom of C_2_H_2_ directly adsorbs above Ge and Cr atom. The adsorption energies of H_2_ and C_2_H_2_ molecules absorbed on GeG system are −0.117 eV and −0.287 eV, and those of the CrG system are −0.390 eV and −1.436 eV, respectively. The results show that CrG system has strong adsorption abilities to H_2_ and C_2_H_2_ molecules, while GeG system is relatively weaker. For CrG system, the charge transfer value of Cr-graphene to C_2_H_2_ (0.079 e) is distinctly higher than that of H_2_ (0.052 e), confirming that the interaction between C_2_H_2_ molecule and Cr-graphene is stronger. In addition, the strong hybridization between atomic orbitals results in the more excellent adsorption performances of CrG to C_2_H_2_. Therefore, the Cr-graphene is expected to be an ideal gas-sensing material for detecting C_2_H_2_ gas.

## Figures and Tables

**Table 1:** The structural parameters of Ge-doped graphene.

Site	$d_{Ge-C}(Å)$	$E_{b}(\mathrm{eV})$	Mulliken Charge (e)
G_T_	1.865	4.725	−0.525
G_H_	2.119	2.301	0.135
G_B_	2.279	1.095	0.032

**Table 2:** The structural parameters of Cr-doped graphene.

Site	$d_{Cr-C}(Å)$	$E_{b}(\mathrm{eV})$	Mulliken Charge (e)
G_T_	1.856	4.732	−0.252
G_H_	2.111	2.031	0.211
G_B_	2.442	1.386	0.236

**Table 3:** The geometrical parameters of H_2_ adsorption systems.

System	d(Å)	$\;E_{ads}(\mathrm{eV})$	$Q_{t}(e)$	Mulliken Charge (e)
IG	H-C 3.026	−0.153	−0.012	C −0.014 H −0.006 H −0.006
GeG	Ge-H 2.908	−0.117	−0.010	Ge −0.529 H 0.005 H −0.015
CrG	Cr-H_1_ 2.030 Cr-H_2_ 1.973	−0.390	0.052	Cr −0.317 H 0.107 H −0.055

**Table 4:** The geometrical parameters of C_2_H_2_ adsorption systems.

System	d (Å)	$\;E_{ads}(\mathrm{eV})$	$Q_{t}(e)$	Mulliken Charge (e)
IG	H-C 2.548	−0.066	−0.008	C_IG_ 0.024 C −0.092 C −0.092 H 0.096 H 0.096
GeG	Ge-C 2.175 Ge-C 2.208	−0.287	0.063	Ge −0.463 C −0.198 C −0.052 H 0.165 H 0.148
CrG	Cr-C_1_ 1.990 Cr-C_2_ 1.981	−1.436	0.079	Cr −0.317 C −0.137 C 0.018 H 0.095 H 0.103

## Data Availability

The data is available on the request from corresponding author.
